# Computational Methods for Molecular Dynamics of Supercooled Water Between 200 and 273 K

**DOI:** 10.3390/e28091029

**Published:** 2026-09-18

**Authors:** Francisco Carrascoza, Konrad Gorzelanczyk, Jacek Blazewicz

**Affiliations:** 1 Institute of Computing Science, Poznan University of Technology, Ul. Piotrowo 2, 61-138 Poznan, Poland; 2 European Centre for Bioinformatics and Genomics (ECBiG), Ul. Piotrowo 2, 61-138 Poznan, Poland; 3 Institute of Bioorganic Chemistry, Polish Academy of Sciences, Ul. Z. Noskowskiego 12/14, 61-704 Poznan, Poland

**Keywords:** AIMD, molecular dynamics, supercooled water, NQE, nuclear quantum effects, density functional theory, path-integral molecular dynamics, PIMD, BOMD

## Abstract

Water exhibits anomalous thermodynamic and structural behaviour as it approaches and crosses below its melting point. Modelling this behaviour computationally remains challenging: as temperature decreases, nuclear quantum effects (NQEs) become increasingly significant, sampling efficiency deteriorates, and the choice of computational method critically impacts the accuracy of predicted structural and dynamical properties. This review aims to provide practitioners with a practical guide to performing reliable molecular dynamics simulations of water at low temperatures, with emphasis on the supercooled regime (200–273 K). We examine commonly used *ab initio* molecular dynamics (AIMD) methods and critically evaluate strategies for incorporating nuclear quantum effects through path-integral molecular dynamics (PIMD), ring-polymer MD (RPMD), and centroid MD (CMD). On cooling through the supercooled regime, the relative importance of nuclear quantum effects increases steadily, so that the error incurred by treating the nuclei classically grows and affects the structural and dynamical properties of interest. The known limitations of density functional approximations for water’s structure are assessed in the context of their interplay with NQE treatment, rather than in isolation. By discussing practical considerations, including sampling efficiency and the temperature scaling of path-integral bead counts, alongside the methods themselves, this review is intended to serve as a reference for reliable AIMD studies of supercooled water.

## 1. Introduction

Water is arguably the most studied liquid in science, yet its behaviour continues to reveal surprising complexity, particularly at low temperatures. The thermodynamic and transport properties of water deviate increasingly from simple-liquid behaviour on cooling, with the deviations already apparent below the temperature of maximum density (∼277 K) and increasing upon supercooling below the equilibrium melting point ($T_{m}$ = 273 K) [1,2]. These anomalies (i.e., density, isothermal compressibility, and heat capacity changes) have motivated decades of theoretical and experimental investigation [3,4,5].

Understanding water’s behaviour at low temperatures is not merely of academic interest. For example, supercooled water plays critical roles in atmospheric chemistry, where it exists metastably in clouds at temperatures as low as 235 K [6]. In biomedical sciences, supercooling preservation enables extended storage of organs, tissues, and cells for transplantation [7,8,9]. In food technology and material processing, controlling supercooled water states is essential for cryopreservation without ice-crystal damage [10]. It also acts as a solvent for complex organic molecule (COM) formation [11,12,13,14]. In planetary science, water ice in various amorphous and crystalline forms is found on icy moons, comets, and Mars [15]. These diverse applications demand accurate theoretical descriptions, aimed at facilitating in silico experimentation, which has important advantages over wet-lab experimentation, such as cost, time and reproducibility.

Classical molecular dynamics (MD) simulations have provided valuable insights into water’s behaviour, but they fundamentally cannot capture several phenomena critical to low-temperature systems. Chemical reactions, including the radical chemistry relevant to astrochemical environments, require explicit treatment of electronic structure. Reactivity is not, however, the exclusive province of electronic-structure methods: reactive bond-order potentials such as ReaxFF form and break covalent bonds within an empirical framework, at a small fraction of the cost [16]. These are the appropriate tool when covalent rearrangement is itself the target; the continual making and breaking of hydrogen bonds that governs the structural behaviour reviewed here is already captured by non-reactive models, and since our concern is the equilibrium and supercooled structure and dynamics of intact water molecules, reactive potentials are not considered further. Separately, the rigid non-polarisable classical water force fields neglect both electronic polarisability and nuclear quantum effects, which limits their accuracy for structural and thermodynamic properties of water at low temperatures [17].

Ab initio molecular dynamics (AIMD) simulations at low temperatures face their own substantial challenges. First, the dynamics slow as temperature decreases, making adequate sampling increasingly difficult within accessible simulation timescales. Second, nuclear quantum effects (NQEs), such as zero-point energy, tunnelling, and proton delocalization, become progressively more important as the thermal energy $k_{B}T$ becomes comparable to vibrational zero-point energies. At 300 K, the ratio of hydrogen zero-point energy to thermal energy is about an order of magnitude, which increases when the temperature drops [18], indicating that classical treatment of nuclei introduces significant systematic errors. Third, the accuracy of different density functionals for water remains a subject of ongoing investigation, with different approximations yielding qualitatively different predictions for structure and dynamics [19].

This review addresses these challenges systematically. We aim to provide practitioners with a guide to performing reliable AIMD simulations of water in the 200–273 K temperature range. Section 2 briefly reviews the structural characteristics of water at low temperatures, establishing the physical context and validation targets for simulations. Section 3 presents the principal AIMD approaches, from Born–Oppenheimer MD (BOMD) to path-integral MD (PIMD), and discusses how each addresses the specific challenges that water simulations face in the supercooled regime; it also covers method selection, sampling, convergence, and validation targets. Section 4 addresses density functional selection for water. Section 5 addresses the central methodological challenge: nuclear quantum effects, the structural consequences, and their interplay with density functional selection.

**Figure 1:**
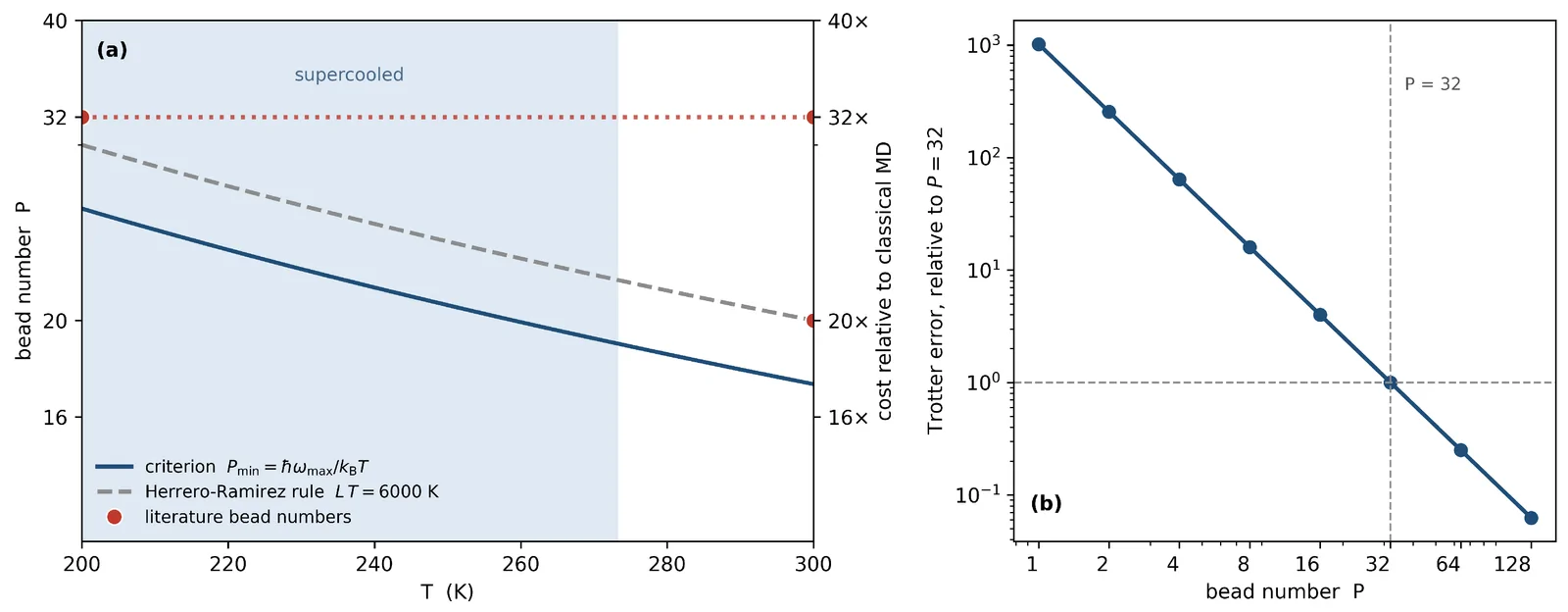
Bead-number convergence for path-integral simulations of water. (**a**) Minimum bead number *P* vs. temperature. Blue solid line: *P* from the convergence criterion $P≳\hbar \omega_{\max}/k_{B}T$, with $\omega_{\max}=\omega_{\mathrm{OH}}=3600$ cm^−1^. Dashed line: constant-precision prescription LT=6000 K (Ref. [51]). Red markers: bead numbers reported in the literature at a stated temperature: P≥32 at room temperature (Ref. [18]); P≥20 at room temperature (Ref. [51]). Dashed red segment: temperature range with P≥32 used in Ref. [52]. Shaded region: the supercooled window. The solid line is derived analytically from the convergence criterion; the dashed line is an empirical fit for constant precision. (**b**) Trotter error vs. bead number, normalised to P=32 and computed by the authors from the $O(P^{-2})$ scaling of Ref. [53]. The absolute prefactor is not available from the source data.

**Figure 2:**
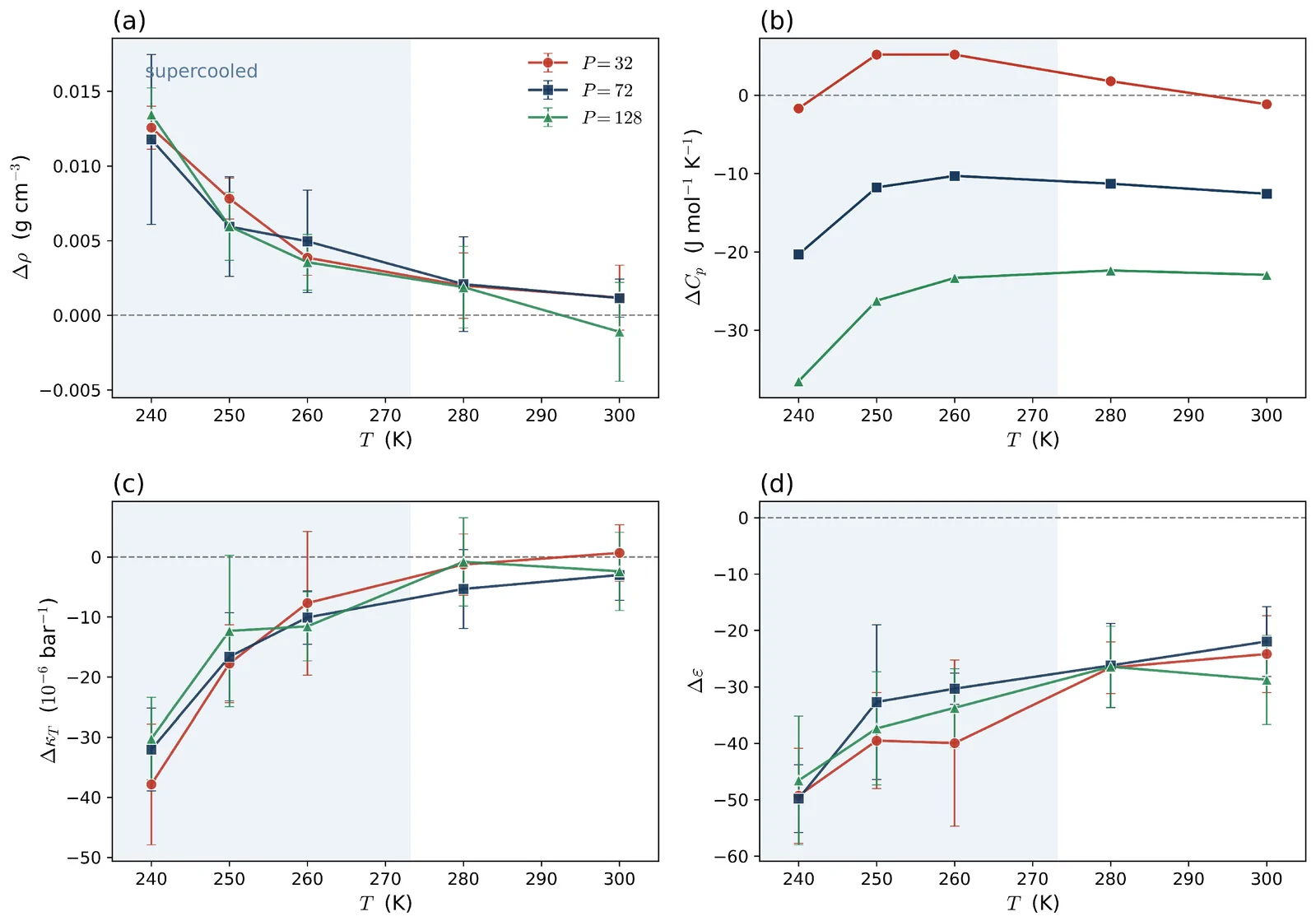
Temperature dependence of bead-number sensitivity in PIMD simulations of q-TIP4P/F water. Deviations from IAPWS reference values (simulation − reference) are shown for (**a**) density ρ, (**b**) isobaric heat capacity $C_{P}$, (**c**) isothermal compressibility $\kappa_{T}$, and (**d**) static dielectric constant ε. Markers: Bead numbers P=32, 72, and 128, all at a time step dt=0.25 fs. A positive ordinate means that the simulation exceeds the reference; the horizontal line at zero marks exact agreement. Error bars are the uncertainties of the simulated series; none are available for $C_{P}$. Simulation values are digitized from Ref. [52]. Reference values are computed at 1 bar and at the exact simulation temperatures, from the IAPWS G12-15 equation of state [55] for ρ, $C_{P}$, and $\kappa_{T}$, and from the IAPWS R8-97 formulation [56] for ε.

**Figure 3:**
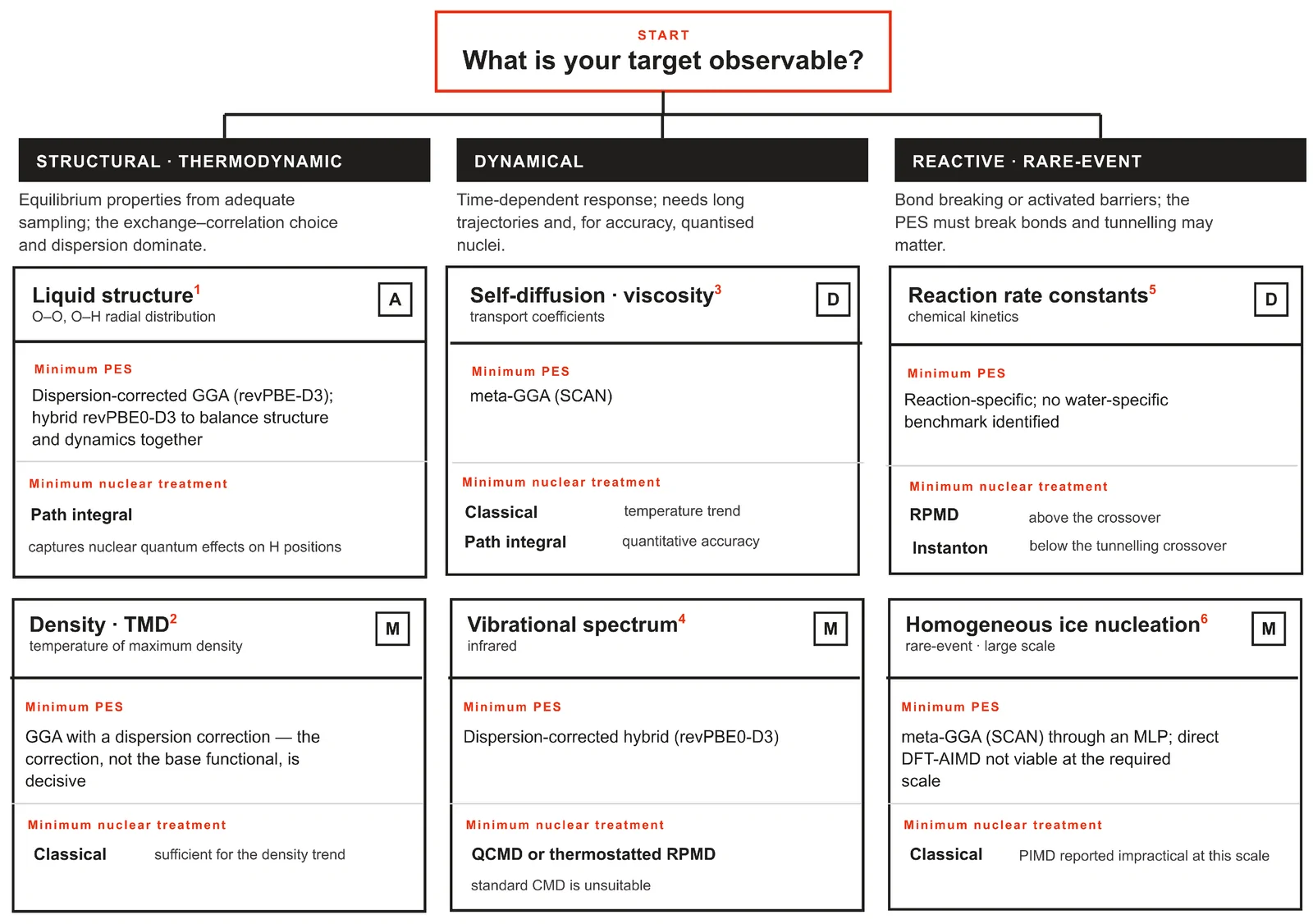
Method-selection map: least demanding PES and nuclear treatment for each target observable. Evidence class of each entry: **D**, direct comparison at the stated state point; **M**, model-dependent result obtained with a single potential or functional; **A**, tested only at ∼300 K, not below 273 K. Branches are numbered 1–6 and keyed to the quantitative basis, state point, and source given in the notes for this figure in the main text.

Other notable reviews exist on functionals, machine-learning potentials, path-integral spectroscopy, and nuclear quantum effects [19,20,21,22]. Our work focuses on the 200–273 K temperature window, where those methodological choices become coupled and the supporting evidence is thinnest. Within this work, we present current evidence of the impact of these choices and how the temperature may (or may not) affect them: analyses of how classical and quantum nuclei methods drift from experimental data for common observables; how, in PIMD, the bead number, cost, and discretisation error scale on cooling (Figure 1 and Figure 2); a method-selection map organised by the target observable (Figure 3); and sampling and validation checklists tied to low-temperature observables (Tables 3 and 4).

## 2. Water’s Structure at Low Temperatures

First, it is essential to establish the physical phenomena that simulations must capture. This section reviews the structural evolution of water from ambient conditions through the 200–273 K supercooled regime.

### 2.1. Supercooled Liquid Water

Supercooled liquid water is a metastable state that has been reached experimentally down to its homogeneous freezing limit of approximately 235 K in micrometre-sized samples [23], and to even lower temperatures using rapidly cooled nanodroplets [24] and ultrafast X-ray probing of evaporatively cooled microdroplets [25], the latter revealing metastable bulk liquid water down to 227 K.

The structure of supercooled water evolves continuously from ambient conditions, with an accelerating increase in ordering as temperature decreases. X-ray diffraction studies reveal that, upon cooling, the first oxygen–oxygen radial distribution function (RDF) peak near 2.8 Å grows in height, while the running O–O coordination number exhibits a temperature-independent isosbestic point near 3.3 Å, and the second peak near 4.5 Å becomes increasingly pronounced below the compressibility-minimum temperature, reflecting enhanced tetrahedral coordination [26]. Measurements extending to 234.8 K show that intermediate-range correlations continue to strengthen upon supercooling, with the 4th and 5th peaks near 9 and 11 Å increasing in height and the 4th-peak position shifting to shorter distances [27]. At ambient conditions, water already exhibits nanometre-scale density fluctuations representing tetrahedral-like and hydrogen-bond-distorted structures [28]. These structural changes are often interpreted within a two-state framework, where water is viewed as a fluctuating mixture of locally tetrahedral, low-density liquid (LDL) and more disordered, high-density liquid (HDL) structural motifs [2]. More recent experiments reported the observation of a liquid–liquid transition under pressure at 205 ± 10 K [29]. The temperature range between approximately 160 K and 232 K, often called the “no-man’s land”, has historically been inaccessible to bulk experiments due to rapid crystallisation [25]. Our understanding of water’s structure below the homogeneous nucleation temperature ($T_{H}\approx 235$ K)—whether it reflects continuous structural evolution or a discrete liquid–liquid transition—continues to evolve, and computational simulations provide much of the structural insight into this experimentally challenging region.

### 2.2. Crystalline Ice Phases

Ice I_*h*_ is the thermodynamically favoured phase throughout the 200–273 K temperature range at ambient pressure, and this is the phase whose formation defines the homogeneous nucleation temperature $T_{H}$ discussed above; Table 1 summarizes its structural properties, together with those of related metastable and higher-pressure phases, as reference points for the liquid-state simulations discussed in this review.

Ice I_c_ is the metastable cubic form of ice [36]. In practice, what has often been reported as “cubic ice” is typically stacking-disordered ice I_sd_, containing cubic sequences interlaced with hexagonal sequences [36], which results in variable density and $r_{\mathrm{OO}}$.

X-ray diffraction measurements on freezing micron-sized water droplets show a droplet-size dependence, in which smaller droplets freeze predominantly to cubic ice with stacking faults while larger droplets favour hexagonal ice [39].

## 3. Ab Initio Molecular Dynamics Methods for Water

The theoretical description of atomic and molecular systems based on the Schrödinger equation emerged from early theoretical developments of electron and nuclear behaviour. The molecular Hamiltonian of the Schrödinger equation comprises terms for nuclear kinetic energy, electronic kinetic energy, electron–nuclear attraction, electron–electron repulsion, and nuclear–nuclear repulsion. Solving this equation exactly for all but the simplest systems presents formidable computational challenges, given that the exact mathematical form of the wavefunction is unknown. Thus, there are some common approximation methods that address the tradeoff between resource consumption and accuracy. This section presents the principal AIMD methods used for water simulations in cold regimes, emphasizing the fundamental origin of their limitations or advantages.

### 3.1. Classical-Nuclei Methods

#### 3.1.1. Born–Oppenheimer Molecular Dynamics (BOMD)

In BOMD, the electronic structure is converged self-consistently at each nuclear configuration, yielding forces from the ground-state energy surface [40]. Nuclei evolve according to Newton’s equations:

(1) $$M_{I}{\ddot{R}}_{I}=-\nabla_{R_{I}}E_{0}\left[\left\{R\right\}\right]$$

where $M_{I}$ and $R_{I}$ are the mass and position of nucleus *I*, and $E_{0}$ is the ground-state electronic energy.

BOMD provides a clean separation of electronic and nuclear timescales. With sufficiently tight self-consistent-field convergence at each step, it conserves the physical total energy [40].

However, because the nuclei are propagated classically, BOMD is unable to capture zero-point energy or nuclear tunnelling; errors grow as *T* decreases and the de Broglie thermal wavelength Λ increases.

BOMD is implemented in CP2K [41], VASP [42], and Quantum ESPRESSO [43] software packages. The Gaussian and augmented plane waves (GPW) method in CP2K offers favourable scaling for 100–500 molecule systems [44].

#### 3.1.2. Car–Parrinello Molecular Dynamics (CPMD)

CPMD avoids explicit self-consistent field (SCF) convergence by treating electronic wavefunctions as dynamical variables with fictitious mass μ [45]:

(2) $$\begin{array}{cc} M_{I}{\ddot{R}}_{I} & =-\nabla_{R_{I}}E\left[\left\{\psi \right\},\left\{R\right\}\right] \end{array}$$

(3) $$\begin{array}{cc} \mu {\ddot{\psi }}_{i} & =-\frac{\delta E}{\delta \psi_{i}^{*}}+\sum_{j}\Lambda_{ij}\psi_{j} \end{array}$$

where $\Lambda_{ij}$ represents a matrix of Lagrange multipliers used to enforce the mathematical constraint of orthonormality.

The method’s validity rests on adiabatic decoupling: the fictitious electronic frequency $\omega_{e}\propto \sqrt{E_{\mathrm{gap}}/\mu }$ must greatly exceed nuclear frequencies $\omega_{n}$, keeping electrons near the ground state. For water, two problems arise at low temperatures. The O-H stretching frequency (∼3600 cm^−1^ at room temperature) sets a high $\omega_{n}$ that constrains the choice of μ, requiring small time steps of ∼0.12–0.24 fs [46,47]. As nuclear motion slows with decreasing *T*, maintaining the ratio $\omega_{e}/\omega_{n}$ becomes harder: small perturbations can transfer energy from electrons to nuclei, causing drift from the Born–Oppenheimer surface [47].

Standard CPMD with classical nuclei is generally not recommended for water when nuclear quantum effects are required. Classical first-principles water is over-structured relative to experiments at room temperature. However, including nuclear quantum effects softens the radial distribution functions and systematically improves agreement with experiments [48]. For low-temperature water, both limitations remain: classical nuclei become a progressively worse approximation as the de Broglie wavelength of the proton grows, making methods with explicit NQEs the more appropriate choice.

### 3.2. Path-Integral Molecular Dynamics (PIMD)

Classical treatment of nuclei fails for light atoms at low temperatures. PIMD addresses this by exploiting the isomorphism between quantum statistical mechanics and classical mechanics of ring polymers [49,50]: each quantum particle of mass *M* is represented by *P* classical “beads” connected by harmonic springs, with spring constant $k_{P}=M\omega_{P}^{2}=MP^{2}k_{B}^{2}T^{2}/\hbar^{2}$, where $\omega_{P}=Pk_{B}T/\hbar$. The temperature dependence of $k_{P}$ is the central feature: at low *T*, the springs weaken (quadratically), allowing the ring polymer to “open” and directly represent the growing thermal de Broglie wavelength of the quantum particle.

Convergence to the quantum limit requires $P≳\beta \hbar \omega_{\max}$, where $\omega_{\max}$ is the highest physical frequency in the system [18]. For water at room temperature, $\omega_{\max}\approx \omega_{\mathrm{OH}}\approx 3600$ cm^−1^, P≈32 replicas are typically required to converge structural properties, and since standard PIMD evaluates forces independently for each bead, computational cost scales linearly with *P* [18]. Since *P* scales as $\hbar \omega_{\max}/k_{B}T$, the requirement grows roughly as 1/T at fixed $\omega_{\max}$. Rescaling the room-temperature value P=32 by this factor gives P=48 at 200 K, which stays above the bare criterion of P=26 by the same margin that it does at 300 K (see Figure 1a), and places standard ab initio PIMD several tens of times more expensive than classical BOMD.

#### 3.2.1. Bead Number, Cost, and Convergence Error on Cooling

The criterion $P≳\beta \hbar \omega_{\max}$ can be evaluated directly over the range of interest. With $\omega_{\max}=\omega_{\mathrm{OH}}\approx 3600$ cm^−1^, i.e., $\hbar \omega_{\max}/k_{B}=5179.6$ K, the bound rises from P≈17 at 300 K to P≈26 at 200 K (Figure 1). Because standard PIMD evaluates forces independently on every bead, the same curve read on the right-hand axis gives the cost relative to classical molecular dynamics: cooling from 300 K to 200 K raises the minimum cost by a factor of 1.50 at fixed accuracy. For instance, Markland and Ceriotti describe the converged bead number as a low multiple of $\hbar \omega_{\max}/k_{B}T$ and quote at least P=32 for room-temperature systems containing O–H bonds [18], while Herrero and Ramírez kept the precision of their ice $I_{h}$ simulations roughly constant across temperatures by adjusting the Trotter number *L* (where *L* represents the bead number *P*) to the inverse temperature, L∝1/T, at fixed LT=6000 K; this gives L=20 at 300 K [51]. Both sit above the bare criterion, confirming that $\beta \hbar \omega_{\max}$ is a floor rather than a converged value (see also Figure 1).

The Trotter error in panel (b) of Figure 1 is the discretization error introduced by splitting the imaginary-time path integral into a finite number of beads *P*; it vanishes only in the limit P→∞, where the ring-polymer representation becomes an exact mapping onto quantum statistical mechanics. Because the primitive Trotter factorization underlying standard PIMD is accurate to second order in the imaginary-time slice width, this error decreases as $O(P^{-2})$: doubling *P* reduces the error by a factor of four. That panel plots the scaling on a log–log axis, normalised so that the error at P=32 is set to unity; because the absolute prefactor is not available from the source data, only the relative, scaling-law shape of the curve should be read from the plot, and it should not be used to estimate the size of the error at any given bead number.

What the criterion does not say is how large an error results from holding the bead number fixed as the temperature changes. Figure 2 addresses this question by comparing PIMD simulations with the q-TIP4P/F water model from Ref. [52] against IAPWS reference values for real water. The q-TIP4P/F model is a flexible, non-polarisable four-site empirical point-charge water model in which the intramolecular O–H stretch is represented by a quartic expansion of a Morse potential, and whose parameters were fitted so that path-integral—rather than classical—simulations reproduce the experimental properties of liquid water [54]. It is therefore a good reference for NQE studies, although quantitative conclusions drawn from it remain model-dependent. Figure 2 reports P= 32, 72, and 128 at a common time step of 0.25 fs at 240, 250, 260, 280, and 300 K, for the density, the isobaric heat capacity, the isothermal compressibility, and the static dielectric constant. From it, two contributions can be separated: the deviation common to all three series, which measures the accuracy of model and method against real water; and the residual spread between the series, which measures the sensitivity to bead number alone.

The common deviation is present at every bead number, keeps the same sign in panels (a), (c), and (d) below 280 K, and grows on cooling. The simulated density differs from the reference by about 0.001 g cm^−3^ at 300 K (0.1%) and by 0.012–0.013 g cm^−3^ at 240 K (1.3%); the isothermal compressibility lies 30–$38\times 10^{-6}$ bar^−1^ below the reference at 240 K, closing to within a few units of zero at 300 K; and the static dielectric constant is too low throughout, by 22–29 units at 300 K and by 47–50 at 240 K—that is, by roughly one third and one half of the reference value, respectively. The dielectric deficit is the expected behaviour of a non-polarisable point-charge model and is not a bead-convergence effect.

The residual bead-number dependence is a much smaller effect, and in three of the four panels there is no clear trend visible. For the density, the compressibility, and the dielectric constant, the three series agree with one another within their combined uncertainties at every temperature plotted, and their ordering with the number of beads is not preserved from one temperature to the next. Given error bars that reach ±0.006 g cm^−3^, $\pm 13\times 10^{-6}$ bar^−1^, and ±14 in ε, no systematic bead-number dependence can be asserted for these three properties between 240 and 300 K. At 300 K, the density and compressibility deviations of all three series are individually consistent with zero within their error bars, whereas the dielectric deviation is not.

The isobaric heat capacity behaves differently. The three series are separated by 22 J mol^−1^ K^−1^ at 300 K, widening to 35 J mol^−1^ K^−1^ at 240 K, and the ordering P=32>72>128 holds at all five temperatures. The P=32 series remains within ±6 J mol^−1^ K^−1^ of the reference and has the smallest deviation of the three, while the P=72 and P=128 series lie below the reference at every temperature. Two observations follow from these results: First, the source data provide no uncertainties for $C_{P}$, so the separation cannot be tested against error bars; the fact that the ordering is monotonic in *P* and is reproduced at all five temperatures argues against random scatter but does not by itself establish significance. Second, agreement with the reference degrades as *P* increases, which is the opposite of what a residual Trotter error alone would produce, and suggests instead a partial cancellation between model error and incomplete bead convergence for this property. The authors argue that $C_{P}$ is sensitive to the simulation time step and to the number of beads *P*. They recommend further studies to determine better parameters for this convergence [52]. The practical consequence for low-temperature work is that there is no guarantee that the convergence parameters established for one observable will carry over to another. Furthermore, within the resolution of the available data, the density, the compressibility, and the dielectric constant tolerate P≈32 down to 240 K, while $C_{P}$, a second derivative of the free energy, obtained in practice by differentiating H(T), is clearly affected by the number of beads used. Regardless of the correct parameterisation of the simulation, the sensitivity of $C_{P}$ is greater on cooling. Eltareb et al. note that, below 240 K, $C_{P}$ increases sharply, and that this increase has not been correctly reproduced by either quantum or classical treatments, in this study or in the others that they report therein, using flexible or rigid water models [52].

#### 3.2.2. Accelerated Path-Integral Methods

Several approaches reduce PIMD’s computational burden while preserving essential quantum effects:

**Ring-polymer contraction (RPC)** exploits the observation that the physical potential V(R) varies more slowly than the inter-bead springs. Expensive DFT forces can be evaluated on a contracted ring ($P^{\prime }<P$ beads), with projection back to the full ring [57]. This is particularly effective when a reference potential can absorb the rapidly varying forces, leaving a smoothly varying remainder for the contracted ring [18].

**PI-GLE** [53] employs a generalized Langevin equation thermostat with coloured noise to accelerate the convergence of path-integral molecular dynamics in anharmonic systems exhibiting both zero-point energy and tunnelling effects. Unlike white noise, coloured noise mimics the quantum fluctuation spectrum and is designed so that a *P*-replica PIMD simulation converges structural observables in the harmonic limit for any *P*. **PIGLET** [58] extends this scheme with additional constraints on the noise spectrum, so that the quantum kinetic energy estimator also converges in the harmonic limit at small *P*. Together, these approaches achieve converged NQEs with as few as 6 beads at 300 K for liquid water [58] and 8 beads at 230 K [59].

Ring-polymer contraction reduces the number of replicas on which the expensive component of the potential is evaluated, which results in a smaller force-evaluation cost [57], whereas PI-GLE and PIGLET instead reduce the number of beads required for convergence itself. The key insight underlying PI-GLE and PIGLET is that quantum statistics require a mean energy per mode of $\frac{1}{2}\hbar \omega \coth\left(\beta \hbar \omega /2\right)$ rather than the classical equipartition value $k_{B}T$; the GLE thermostat enforces this distribution while reducing the number of beads required to converge structural and momentum-dependent estimators, respectively. Both are implemented in the i-PI software package [60], which interfaces with CP2K and other electronic-structure codes.

Of these two routes, only the bead-number reduction of PI-GLE and PIGLET yields a saving effective on cooling, since the number of beads required for convergence scales as $P≳\beta \hbar \omega_{\max}$. Although these methods reduce the cost of converging the quantum description of the nuclei, reaching a given trajectory length remains more expensive than in standard AIMD, so the configurational sampling required for ergodicity across the basins of the free-energy landscape is correspondingly more costly. Direct simulation of phenomena such as glass formation, nucleation, or phase transformations remains computationally demanding due to the cost of sampling.

Enhanced sampling addresses the sampling inefficiency problem directly: by biasing the dynamics in a controlled manner, these methods accelerate convergence toward ergodic sampling, making it possible to recover energy barriers and rates that lie beyond the reach of the accessible trajectory length. Enhanced sampling is a large and continuously evolving field, and we list here only a few representative methods. Umbrella sampling [61] and metadynamics [62] bias the sampling along chosen collective variables to reconstruct free-energy surfaces; metadynamics with collective variables designed for crystallisation has been used to drive reversible liquid–ice transitions in the TIP4P/Ice model and to estimate nucleation rates at several undercoolings [63]. For nucleation rates specifically, two complementary routes exist: the seeding technique inserts a crystalline cluster and locates the size at which it is equally likely to grow or melt, and then combines that critical size with classical nucleation theory [64], an approach used in the ab initio machine-learning study of Ref. [65]; forward-flux sampling [66] instead computes the rate directly from ensembles of partial trajectories without assuming classical nucleation theory, and a temporally coarse-grained variant provided the first direct homogeneous ice nucleation rate for a molecular water model, TIP4P/Ice at 230 K and 1 bar [67]. A comprehensive description or listing of these methods is outside the scope of this work; we refer the reader to a recent review of the field [68].

### 3.3. Approximate Quantum Dynamics in Real Time

Imaginary-time path integrals give the quantum statistics of the nuclei, but the ring-polymer trajectory is not itself a real-time trajectory; time correlation functions, vibrational spectra, and rate constants therefore require a further approximation.

Ring-polymer molecular dynamics (RPMD) approximates Kubo-transformed real-time correlation functions by evolving the ring polymer under its own classical Hamiltonian, which preserves the exact quantum Boltzmann distribution and reduces to classical molecular dynamics in the high-temperature limit [69,70]. It has been applied to chemical reaction rates [71] and to transport coefficients [70]. Its characteristic artefact is the appearance of spurious resonance features in vibrational spectra, produced by the internal modes of the ring polymer; thermostatted RPMD (TRPMD) suppresses them by attaching a Langevin thermostat to those internal modes [72].

Centroid molecular dynamics (CMD) rests on the Feynman path-centroid formulation of quantum statistical mechanics [73], in which the centroid of the ring polymer is propagated on the potential of mean force generated by the remaining modes [21]. For quantum liquid water, this produces an artificial red shift and broadening of the O–H stretching band; the artefact originates in the curvature of the centroid potential of mean force, is known as the curvature problem, and becomes more severe as temperature falls [74]. Quasi-centroid molecular dynamics (QCMD) removes it by defining the centroid in curvilinear rather than Cartesian coordinates [75].

Below the crossover temperature at which tunnelling replaces thermally activated barrier crossing, ring-polymer instanton theory obtains rate constants from the dominant tunnelling path rather than from real-time propagation [76]. The state points at which each of these methods has been tested are given in Section 3.5.

### 3.4. Machine-Learning Interatomic Potentials

Machine-learning interatomic potentials (MLPs), including neural-network potentials and kernel-based methods such as Gaussian Approximation Potentials, are trained on a database of ab initio (typically DFT) energies and forces and then used as a drop-in replacement for the underlying potential energy surface during MD [77,78]. Once trained, force evaluations cost a small fraction of a DFT step. They can be combined with BOMD, PIMD, PIGLET, RPMD, or CMD, and the speedup applies in each case. In the path-integral context in particular, the *P*-fold overhead of bead sampling becomes affordable, and nanosecond-scale path-integral trajectories of condensed-phase water at near-DFT accuracy are now feasible [79].

A comprehensive review of MLPs for water is beyond the scope of this work; we refer the reader to a recent dedicated review on neural-network potentials [20] and note that data-driven quantum simulations have recently reproduced the water phase diagram at near-first-principles accuracy [80]. We note one caveat directly relevant to the present review: the great majority of MLPs for water reported to date have been trained and validated on reference data sampled at or near ambient conditions. Their transferability to the supercooled regime is not guaranteed and remains an active research question. Studies that use MLPs for low-temperature water should therefore include explicit validation against experimental values or ab initio reference points at the temperatures and densities of interest, rather than relying on extrapolation from ambient-condition training sets.

### 3.5. Comparison of AIMD Methods and Method Selection

Method selection depends on the target observable, the required accuracy, and the temperature regime, rather than on a single criterion. Table 2 summarizes these tradeoffs together with the parameter to converge or check for each method. Each method has several parameters requiring convergence; Table 2 lists only the one that matters most for water simulations. Broadly, classical-nuclei AIMD is appropriate when NQE corrections to the target property are smaller than other modelling errors; path-integral methods (PIMD, PIGLET) are needed when quantum statistics of the nuclei matter, with the number of beads *P* growing as *T* decreases; and approximate quantum-dynamics methods (RPMD, CMD/QCMD, instanton) are needed when real-time correlation functions, vibrational spectra, or rate constants are required. The boundaries between these regimes are not sharp and should be assessed against benchmarks for the specific property of interest.

Figure 3 offers a method-selection map: for each of the observables most commonly reported in this temperature range, it gives the least demanding PES and the least demanding nuclear treatment for which a published study demonstrates agreement with experiments or with a higher-level theoretical reference. The quantitative basis, state point, and source for each branch, keyed to the numbering in the figure, are given below.

**Notes on Figure 3**

1. *Liquid Structure (O–O, O–H RDF):* Classical-nuclei revPBE-D3 reproduces the experimental O–O RDF at 300 K and 1 atm, and NQEs shift it only mildly with revPBE-D3 or negligibly with revPBE0-D3 functionals; classical dynamical quantities such as the diffusion coefficient, however, carry >20% statistical error from ∼20 ps trajectories, which path-integral runs of >700 ps (revPBE-D3) and >300 ps (revPBE0-D3) reduce below 5% [82].
2. *Density; Temperature of Maximum Density:* Neural-network potentials at 1 bar: TMD 256 K, ρ=1.054 g cm^−3^ (BLYP-D3) and 274 K, ρ=0.901 g cm^−3^ (RPBE-D3); no density maximum without dispersion. Experiment: 277.13 K, ρ=0.99997 g cm^−3^. Deviation from the experimental density 5–10% with dispersion, 20–40% without [84].
3. *Self-Diffusion; Shear Viscosity:* Among SCAN, PBE-D3, and optB88-vdW, SCAN best reproduces the temperature evolution of the shear viscosity and self-diffusion coefficient but overestimates the viscosity by more than a factor of 1.7 between 260 and 300 K relative to a TIP4P/2005 reference. NQE assessed by PIMD at a limited set of temperatures [85].
4. *Vibrational Spectrum (IR):* q-TIP4P/F ice Ih at 150 K: the CMD O–H stretch is artificially red-shifted by more than 100 cm^−1^ and broadened, and the TRPMD stretch is severely broadened, whereas the QCMD stretch is sharp [75]. In the 300 K liquid, the CMD stretch is red-shifted by ∼50 cm^−1^ relative to TRPMD [74,75]. QCMD costs approximately 2, 8, and 32 times partially adiabatic CMD at 600, 300, and 150 K [75].
5. *Reaction Rate Constants:* Gas-phase H + CH_4_, 225–400 K: RPMD rate coefficients lie within a factor of 2 of exact quantum results in the deep-tunnelling regime. No condensed-phase water benchmark of comparable rigour was identified [71,86].
6. *Homogeneous Ice Nucleation:* SCAN-trained MLP, seeding, up to $3\times 10^{5}$ atoms at 1 atm. Melting point 312 K against ∼273 K in experiment. Ice Ih–water interfacial free energy $\bar{\gamma }=36\left(2\right)$ mJ m^−2^ against 29.1(8) mJ m^−2^ in experiment; |Δμ| underestimated by 9% at 35 K supercooling, against 17% for TIP4P/Ice and 1% for mW. Nucleation rates agree with experiments within the stated calculation uncertainty; NQE neglected [65].

Three features of Figure 3 deserve emphasis: First, the entries are not interchangeable in evidential status, and the figure labels them accordingly—some rest on a direct comparison performed at the stated state point, others are model-dependent results obtained with one particular potential or functional, and others have only been performed at ambient conditions and, to the best of our knowledge, have never been tested below 273 K. Second, quantitative deviations are reported wherever the primary source states one, and they are marked as not reported otherwise. Third, the coverage is markedly uneven: the density anomaly, the vibrational spectrum, and ice nucleation have been examined with explicit low-temperature reference points, whereas the entries for liquid structure rest on ambient-temperature evidence only, which is a direct consequence of the sparse validation of any functional in the 200–273 K window noted in Section 4. The map is therefore a summary of what has been demonstrated, offered as a guide rather than a prescription; as with Table 2, the boundaries between branches are soft and should be re-examined against benchmarks for the specific property of interest.

### 3.6. Practical Sampling and Convergence at Low Temperature

The methods of Section 3.1, Section 3.2, Section 3.3 and Section 3.4 control how accurately the forces and the nuclear statistics are represented; they do not control whether a trajectory is long enough for the configurations that it visits to be representative. On cooling, this second question becomes the limiting one. In classical models of supercooled water, the α-relaxation time, which represents the timescale over which the local structure decorrelates from its initial configuration, grows steeply as temperature decreases. This relaxation time crosses over from mode-coupling to Arrhenius behaviour in the deeply supercooled region [87,88]. In other words, the temperature dependence of the relaxation time changes character in the supercooled region. Path-integral simulations spanning 200–300 K have been reported with empirical flexible potentials [52], but the trajectory lengths affordable with ab initio forces are shorter by orders of magnitude. Equilibrium sampling in this regime therefore cannot be assumed; it must be demonstrated for the property of interest.

Table 3 lists a selected set of specific diagnostics to ensure proper sampling. Note that each method demands particular diagnostic tests not listed here. Two points deserve emphasis: First, the uncertainty on a time average must be obtained by block averaging or an equivalent treatment of correlated data, because the naive standard error underestimates it [89]. Second, crystallisation competes with equilibration in this temperature range but does not necessarily preclude it: in transiently heated films, the structural relaxation time was found to be at least ten times—and typically more than a hundred times—shorter than the crystallisation time [90]. The liquid can therefore be equilibrated, provided the run is monitored for incipient ice rather than assumed to remain amorphous. We have refrained from introducing numerical values in Table 3. Criteria are stated relative to quantities measured from the simulation itself, because no published protocol fixes equilibration times, trajectory lengths, cell sizes, or replica counts for water in this temperature range. However, the references listed in this table provide suitable starting points for a given method and set of simulation conditions.

### 3.7. Structural and Thermodynamic Validation Targets

The checks of Section 3.6 establish that a trajectory is converged; they do not establish that it reproduces the structure of water. The two are independent failure modes: a well-equilibrated trajectory on an inadequate potential energy surface converges to the wrong structure. Table 4 lists the observables against which a simulation in this temperature range should be compared, together with the experimental reference available inside or close to the 200–273 K window. Three points govern its use: First, $g_{\mathrm{OO}}\left(r\right)$ alone is not a sufficient structural test—$g_{\mathrm{OH}}\left(r\right)$ and $g_{\mathrm{HH}}\left(r\right)$ are the partials that respond most strongly to the nuclear treatment [79], and all three are available experimentally from neutron diffraction down to 220 K [100]. Second, agreement should be assessed directly in reciprocal space, comparing the simulated structure factor S(Q) against the experimental S(Q), rather than relying on real-space g(r) alone: converting neutron data into site–site radial distribution functions is itself model-dependent [100,101]. Third, ring statistics and intermediate-range correlations of the water structure are used here as diagnostics of the hydrogen-bond network in the supercooled liquid, not to characterise ice geometry; ring counts depend on the counting scheme used, which must therefore be reported [102]. Within this temperature window, the key experimental reference point is the maximum of the isobaric heat capacity, located at 229 K by ultrafast calorimetry on evaporatively cooled microdroplets [103].

## 4. Density Functional Selection for Water

The functional choice is perhaps the most consequential decision in DFT-based AIMD of water. Water presents a stringent test for density functionals because accurate simulation requires simultaneous description of the following:

- Intramolecular covalent bonding (O-H stretching, H-O-H bending);
- Intermolecular hydrogen bonding (electrostatic + charge transfer);
- London dispersion interactions;
- Many-body polarisation effects.

Different classes of functionals handle these interactions with varying fidelity, and errors in any component propagate into structural and dynamical predictions [19]. Below, we discuss these nuances family by family, presenting representative members of each class, including both widely used functionals and those that have performed comparatively well in benchmark studies of liquid water and ice. We note an important caveat: most systematic benchmarks of these functionals have been carried out at or near ambient conditions, including the broad surveys of Gillan et al. [19] and the more recent force-based benchmark of more than 50 functionals by Cui [107]. Direct DFT-AIMD validation of any functional in the supercooled regime remains sparse. No cross-functional quantitative comparison is offered, because we found no published benchmarks that are mutually comparable. Most of what is known about low-temperature water at the DFT level comes indirectly through machine-learning potentials trained on DFT reference data [65,79,83,108], which then enable the much longer trajectories needed to probe supercooled temperatures.

### 4.1. Generalized Gradient Approximation (GGA) Functionals

GGA functionals are computationally efficient but suffer from well-documented limitations for water:

**BLYP** [109,110]: Without dispersion correction, BLYP over-structures liquid water and underestimates its diffusivity [111,112]; the density of the liquid is also significantly under-predicted [112]. Adding an empirical Grimme dispersion correction (BLYP-D in the original Schmidt et al. study, equivalent to modern BLYP-D3 in current practice) significantly improves the predicted density and brings the O–O radial distribution function into close agreement with experiments [112]. The resulting BLYP-D3 potential energy surface is comparatively “soft,” and several authors have argued that this softness partially compensates for the missing nuclear quantum delocalization in classical-nuclei simulations [19]. Validation of BLYP-D3 in the supercooled regime is sparse. However, some structural features (e.g., g(r)O–O) are covered in the 230–350 K range in Ref. [113].

**Advantages:** Fast computation; soft PES partially compensates for missing NQE; good for equilibration.

**Disadvantages:** Bare BLYP underbinds the water dimer (4.18 kcal mol^−1^ vs. 5.44 kcal mol^−1^ experimental) [114] and produces an over-structured liquid with too slow diffusion (∼4× below experiment) [114,115]. Adding D3 brings density and the O–O RDF close to experiments [112], but barrier heights remain unreliable: the GMTKN55 BH76 WTMAD-1 is 5.19 kcal mol^−1^ for BLYP and 5.53 kcal mol^−1^ for BLYP-D3(BJ), reflecting an underlying self-interaction error that dispersion correction does not address [116].

**PBE** [117]: PBE tends to over-structure the radial distribution functions of water even at ambient conditions, predicting too many hydrogen bonds and underestimating diffusivity relative to experiments [114]. Adding dispersion (PBE-D3) improves the structural parameters. However, dispersion correction has limited effect on bulk water structure because PBE already overbinds molecules [19].

**Advantages:** Well-validated; widely available; reasonable lattice constants for ice I_h_ [114].

**Disadvantages:** Over-structures liquid water, an artefact not fully removed by adding dispersion corrections [111,114]; poor dynamics in the supercooled regime [85]; overestimates the melting point of ice [118], which is equivalent to over-stabilizing ice relative to the liquid.

**revPBE-D3** [119,120]: revPBE without dispersion correction performs poorly for condensed-phase water, but combined with Grimme’s D3 dispersion correction it has been reported to describe ambient liquid water (298 K, 1 atm) remarkably well in classical AIMD, with equilibrium density, $g_{\mathrm{OO}}\left(r\right)$, self-diffusivity, IR spectrum, and dipole moment in reasonable agreement with experiments [121]. This performance partly reflects a cancellation between intrinsic functional error and the neglect of nuclear quantum effects in classical-nuclei trajectories [121].

Direct validation in the supercooled regime for this functional is sparse: in the ab initio benchmark of Cheng et al., only the revPBE0-D3 version of this functional was tested [79] (see below), and revPBE-D3 was not among the functionals tested in the ab initio benchmark of Herrero et al., where it is named only as a functional that might perform comparably or better [85]. Consequently, applicability of revPBE-D3 to the 200–273 K regime should be regarded as inferred from ambient-temperature benchmarks rather than directly validated.

### 4.2. Meta-GGA Functionals

Meta-GGA functionals incorporate the kinetic energy density τ, providing additional flexibility:

**SCAN** [122]: The strongly constrained and appropriately normed (SCAN) functional satisfies all 17 known exact constraints for a semi-local functional. For water, SCAN captures the density difference between liquid water and ice I_h_ at ambient conditions and reproduces many structural, electronic, and dynamic properties of liquid water without empirical dispersion corrections [123], and it correctly predicts ice I_h_ as more stable than ice I_c_ along with reasonable melting properties of both polymorphs [124]. However, SCAN overestimates the liquid water density by approximately 5% relative to experiments [123] and can exhibit numerical grid sensitivity in some implementations [125,126].

**Advantages:** No empirical parameters; good ice energetics including correct I_h_/I_c_ stability ordering; captures intermediate-range dispersion.

**Disadvantages:** Over-dense liquid (∼5%); grid sensitivity; computational overhead (1.5–2× GGA); can be unstable in some codes.

**r^2^SCAN** [126]: A regularized version of SCAN with improved numerical stability.

For water, r^2^SCAN-driven AIMD reproduces the structure of bulk liquid water at ambient density when simulations are run at an elevated temperature of 350 K, similar to the compensation required for SCAN, which exhibits excessive structuring at 300 K and an augmented first solvation shell at 330 K [127]. The first O–O peak position of r^2^SCAN (2.865 Å) sits slightly above the experimental value (2.801 Å), indicating mild residual over-structuring [127]. Furness et al. report that r^2^SCAN converges with fewer iterations than SCAN [126].

**Advantages:** Improved numerical convergence over SCAN [126]; comparable structural accuracy for bulk liquid water at ambient conditions [127].

**Disadvantages:** Inherits SCAN’s need for an elevated simulation temperature (∼350 K) to match the 300 K experimental RDF [127]; mild over-structuring of the first O–O peak relative to experiments [127].

**DC-SCAN** (Density-corrected SCAN): Applies a Hartree–Fock density correction scheme to SCAN, minimising density-driven errors and raising the accuracy of the SCAN functional towards that of coupled-cluster theory for water clusters and interaction energies [128].

**Advantages:** Reaches near-coupled-cluster accuracy for water cluster energetics; reduces SCAN’s overbinding/over-structuring tendencies; provides a reliable reference for parameterising many-body and machine-learned potentials.

**Disadvantages:** Not standard in most codes; requires a Hartree–Fock density evaluation in addition to the SCAN energy [128]; additional computational overhead; condensed-phase MD applications rely on derived many-body potentials (e.g., MB-SCAN(DC)) rather than direct AIMD [128].

### 4.3. Hybrid Functionals

Hybrid functionals include a fraction of exact (Hartree–Fock) exchange, improving descriptions of charge transfer and reducing self-interaction error [129].

In the context of liquid water, hybrid functionals combined with nuclear quantum effects (NQEs) have been shown to provide a markedly improved description of hydrogen bonding, with revPBE0-D3 + PIMD identified as one of the few approaches that closely agrees with experiments [82].

**PBE0** [130]: With 25% exact exchange, and when paired with the D3 dispersion correction (PBE0-D3), this functional provides excellent barrier heights for reactions [131,132] and reliable magnetic properties [133]. Under classical nuclei, PBE0-D3 tends to over-structure liquid water [134].

The computational cost is substantially higher than that of GGA due to the evaluation of exact exchange (commonly an order of magnitude or more in conventional Gaussian or plane-wave AIMD implementations), although schemes like the auxiliary density matrix method (ADMM) can reduce this overhead by roughly a factor of 20 [135].

**Advantages:** Excellent barrier heights (mean absolute deviation ∼1–2 kcal mol^−1^ on representative benchmarks) [132]; reliable magnetic properties [133]; reduced self-interaction error [129]; improved hydrogen-bond description, particularly when combined with NQEs [22].

**Disadvantages:** Substantial cost increase over GGAs [135]; tends to over-structure water under classical nuclei [134]; requires dispersion correction (e.g., D3 [120]) for condensed phases [134].

**revPBE0**: Combines the exchange of revPBE with 25% exact exchange. This functional has been shown to accurately reproduce the structure, dynamics, and vibrational spectroscopy of bulk liquid water when combined with path-integral simulations and D3 dispersion [79,82,136], and it is one of the few approaches that yields quantitative agreement with experiments for the O–O radial distribution function, self-diffusion coefficient, and full IR and Raman spectra at ambient conditions [82,136].

**Advantages:** Among the best-performing functionals for bulk liquid water when combined with NQEs, capturing the O–O RDF, diffusion, and IR/Raman spectra in close agreement with experiments [79,82,136]; balanced description of covalent bonding and hydrogen bonding such that competing nuclear quantum effects on the hydrogen bond cancel correctly [82].

**Disadvantages:** Substantial cost overhead from the evaluation of exact exchange in periodic AIMD, although acceleration schemes such as ADMM significantly reduce this cost [135]; NQE treatment essential for quantitative agreement of dynamics and vibrational spectra [82,136].

**ωB97X-V** [137]: A range-separated hybrid GGA with the VV10 non-local correlation functional, designed against a broad training set covering thermochemistry, kinetics, and non-covalent interactions. Its meta-GGA counterpart ωB97M-V [138] extends this design to the meta-GGA rung. On benchmark sets of small water clusters with coupled-cluster reference binding energies, ωB97X-V and ωB97M-V have been reported to give the smallest errors among the DFT functionals tested in that set [139]. A closely related meta-GGA, B97M-rV (B97M-V with the rVV10 non-local correlation [140]), gives bulk liquid water structure, diffusion, and IR spectroscopy of comparable accuracy to the much more expensive hybrid revPBE0-D3 when combined with path-integral simulations [136], and is therefore an attractive meta-GGA-level alternative to hybrid functionals for bulk water.

**Advantages:** Smallest binding-energy errors among DFT functionals tested on water-cluster benchmarks against coupled-cluster references [139]; built-in VV10 non-local correlation, so no separate dispersion correction is required [137,141]; the related meta-GGA B97M-rV reaches near-hybrid accuracy for bulk water at meta-GGA cost [136].

**Disadvantages:** Substantial cost relative to GGA functionals, from range-separated exact exchange combined with VV10 non-local correlation evaluation; less commonly used in condensed-phase AIMD of liquid water than global hybrids such as PBE0 and revPBE0.

### 4.4. Dispersion Corrections

Standard DFT functionals miss long-range dispersion interactions, which contribute significantly to ice cohesion (see [142,143]). Common corrections include the following:

- **D3(BJ)** [120,144]: Damped pairwise $C_{6}/R^{6}$ correction with Becke–Johnson damping; well validated and widely available.
- **D4** [145]: Improved atom-pairwise dispersion with charge-dependent coefficients.
- **VV10** [141]: Vydrov and Van Voorhis non-local correlation functional that captures dispersion from the electron density alone. More physically motivated than atom-pairwise schemes, but computationally more demanding due to the six-dimensional double integral over the density.

For water simulations, D3(BJ) remains the default choice, because of its extensive validation, low computational overhead, and availability in all major codes.

## 5. Nuclear Quantum Effects

### 5.1. Physical Origin of Nuclear Quantum Effects

Because electrons move much faster than nuclei due to their significantly smaller mass, the Schrödinger equation is commonly simplified using the Born–Oppenheimer approximation. This approximation underlies the most widely used methods for molecular dynamics, including BOMD, CPMD, and standard DFT-based approaches. In the Born–Oppenheimer approximation, the electronic wavefunction is solved quantum-mechanically at fixed nuclear positions, separating the electronic and nuclear degrees of freedom. This generates a potential energy surface on which nuclei evolve. A further approximation (treating nuclei as classical point particles obeying Newton’s equations) is applied in standard MD methods. This classical treatment of nuclei provides accurate results with reasonable computational cost under most conditions but inherently neglects nuclear quantum effects (NQEs) such as zero-point energy (the minimum energy a particle possesses even at 0 K) and quantum tunnelling, where a nucleus can traverse a potential energy barrier even when its kinetic energy is classically insufficient. For most systems at ambient conditions, neglecting NQEs introduces minimal error and is therefore an acceptable approximation [40].

Consequently, Equation (1) introduces systematic errors whose relative importance grows with decreasing temperature, as the classical treatment cannot account for proton delocalization or for the zero-point contribution to hydrogen-bond fluctuations [18,22]. The magnitude of the resulting error depends on the observable and the system: in general, it remains small for bulk thermodynamic quantities, for which the intramolecular and intermolecular contributions largely cancel, and may become substantial for quantities that resolve the hydrogen explicitly and for transport coefficients (Section 5.4).

The three principal phenomena of NQEs are as follows:

**1. Zero-Point Energy (ZPE):** Quantum mechanics requires that a particle confined in a potential well possesses a minimum energy $E_{0}=\frac{1}{2}\hbar \omega$ even at absolute zero temperature, where ω is the vibrational frequency.

The importance of these nuclear quantum effects can be quantified by the ratio of zero-point energy to thermal energy, $E_{0}/k_{B}T=\hbar \omega /\left(2k_{B}T\right)$. Table 5 lists this ratio, together with the free-particle thermal de Broglie wavelength $\Lambda_{H}$ of hydrogen, over the range from 298 K down to 263 K. Classical Boltzmann statistics break down once $\Lambda_{H}$ is of the same order as, or larger than, the interatomic spacing [146].

Across the whole range in Table 5, every mode satisfies $\mathrm{ZPE}/k_{B}T>1$, so none of them is thermally populated in the classical sense: the O–H stretch is the most strongly quantised (8.36–$9.48\;k_{B}T$), the bend follows (3.99–$4.51\;k_{B}T$), and the librations are closest to the classical limit (1.73–$2.02\;k_{B}T$). This ordering matters for simulation practice. Classical molecular dynamics assigns $k_{B}T$ of energy per vibrational mode irrespective of ℏω, so the stretch and the bend are left essentially unpopulated relative to their quantum ground state. Between 298 and 263 K, the three ratios rise by 13.4% (stretch), 13.0% (bend), and 16.8% (libration). The libration therefore shows the largest relative change, but the margin over the other two modes is under four percentage points across a 35 K interval, and the librational frequencies in these rows were obtained in the source from an empirical $\tilde{\nu }$–*T* relationship rather than by direct measurement, so this ordering should be read as indicative rather than established.

ZPE values are harmonic estimates, $\frac{1}{2}hc\tilde{\nu }$, evaluated at the frequencies tabulated in each source rather than at directly observed band maxima; because the O–H stretch is strongly anharmonic, these should be read as order-of-magnitude indicators of the quantum–thermal energy balance rather than as spectroscopic zero-point energies.

For the O–H stretching mode in liquid water at 298 K (${\tilde{\nu }}_{\mathrm{str}}=3462$ cm^−1^ [147]), the zero-point energy $E_{0}=\frac{1}{2}hc\tilde{\nu }\approx 4.9$ kcal mol^−1^ is comparable to the hydrogen bond strength itself (∼5 kcal mol^−1^). This ZPE causes hydrogen atoms to be more delocalized than classical mechanics predicts at all temperatures: for the O–H stretch, Ref. [53] gives a root-mean-square proton displacement of ≈0.07 Å that is essentially temperature-independent over this range, because the mode is in its quantum ground state ($\mathrm{ZPE}/k_{B}T\gg 1$). For hydrogen, $\Lambda_{H}$ grows from 1.01 to 1.07 Å over the 298–263 K range of Table 5. At room temperature, $\Lambda_{H}$ is already of the same order of magnitude as the interatomic spacing. NQEs do not simply weaken or strengthen hydrogen bonds; because the O–H stretch potential is anharmonic, they produce competing effects [22,54]:

a. *Quantum Fluctuations along the Covalent Bond Axis* (O–H stretch): Motion along the bond axis extends the proton towards the acceptor oxygen, permitting increased proton sharing that shortens the O⋯H distance and strengthens the hydrogen bond [54].
b. *Quantum Fluctuations Orthogonal to the O–H Axis* (librational and bending modes): Motion in these modes lets the proton spread in the other directions, bending the O–H⋯O arrangement and facilitating hydrogen-bond breaking, weakening the network [22]. In general, the trend is that NQEs make weak hydrogen bonds weaker and strong bonds stronger [148].

The balance between these competing effects is tuned by the hydrogen-bond strength, the temperature, and the particular property considered [18]; the cancellation is therefore not uniform across properties and systems.

**2. Quantum Tunnelling:** Nuclei can traverse potential energy barriers even when their kinetic energy is classically insufficient to surmount them. For light nuclei such as hydrogen, this effect is particularly pronounced: the tunnelling probability depends exponentially on both the barrier width and the particle mass, and the low mass of the proton makes hydrogen transfer reactions especially susceptible.

**3. Exchange Effects:** In principle, identical nuclei should obey quantum exchange statistics (Bose–Einstein or Fermi–Dirac). However, in molecular water and ice, protons are localized in deep covalent potential wells, which exponentially suppresses the inter-molecular wavefunction overlap required for exchange. In standard path-integral simulations of water, nuclei are treated as distinguishable particles [18], and this approximation is considered valid at all temperatures relevant to condensed-phase water, in contrast to weakly bound systems such as superfluid helium where exchange effects dominate [149]. Bosonic-exchange path-integral methods do exist [150] but are unnecessary in this regime. Exchange effects are therefore not considered further in this review.

### 5.2. Isotope Substitution as an Experimental Probe of NQE

Nuclear quantum effects manifest experimentally as isotope effects: replacing H with D (or ^16^O with ^18^O) alters the zero-point energy and, hence, the measured structure, dynamics, and thermodynamics; this makes the H_2_O/D_2_O comparison the principal experimental route for validating quantum-nuclei simulations [22]. The competing intra- and intermolecular contributions introduced above (Section 5.1) are central to interpreting these data. Although the underlying changes are sizeable, with the O–H covalent bond in H_2_O reported to be up to ∼3% longer than the O–D bond in D_2_O, and the corresponding intermolecular hydrogen bond ∼4% shorter [22], the net effect on the donor–acceptor separation is far smaller. The *secondary* geometric isotope effect—that is, the change in the distance between two oxygens upon H/D substitution—amounts to only ∼0.001 Å in liquid water and ice I_h_, which is taken as evidence that the competing quantum effects almost exactly cancel under these conditions [22]. The *primary* geometric isotope effect, by contrast, refers to the change in the O–H bond length itself, and its experimental magnitude remains uncertain: analyses based on oxygen substitution and a different analysis technique place the covalent-bond change upon deuteration nearer 0.5% [22].

Such near-cancellations are not universal: water’s isotope effects span a wide range across properties, from very large to almost negligible depending on the property and the thermodynamic conditions [18]—a dichotomy that the competing-quantum-effects principle rationalises. Because reproducing this balance requires an accurate description of a broad region of the potential energy surface, isotope-resolved observables (geometries, proton kinetic energies, and fractionation ratios) provide a stringent simultaneous test of both the electronic-structure method and the nuclear treatment [22].

### 5.3. Structural Consequences of NQE

**Figure 4:**
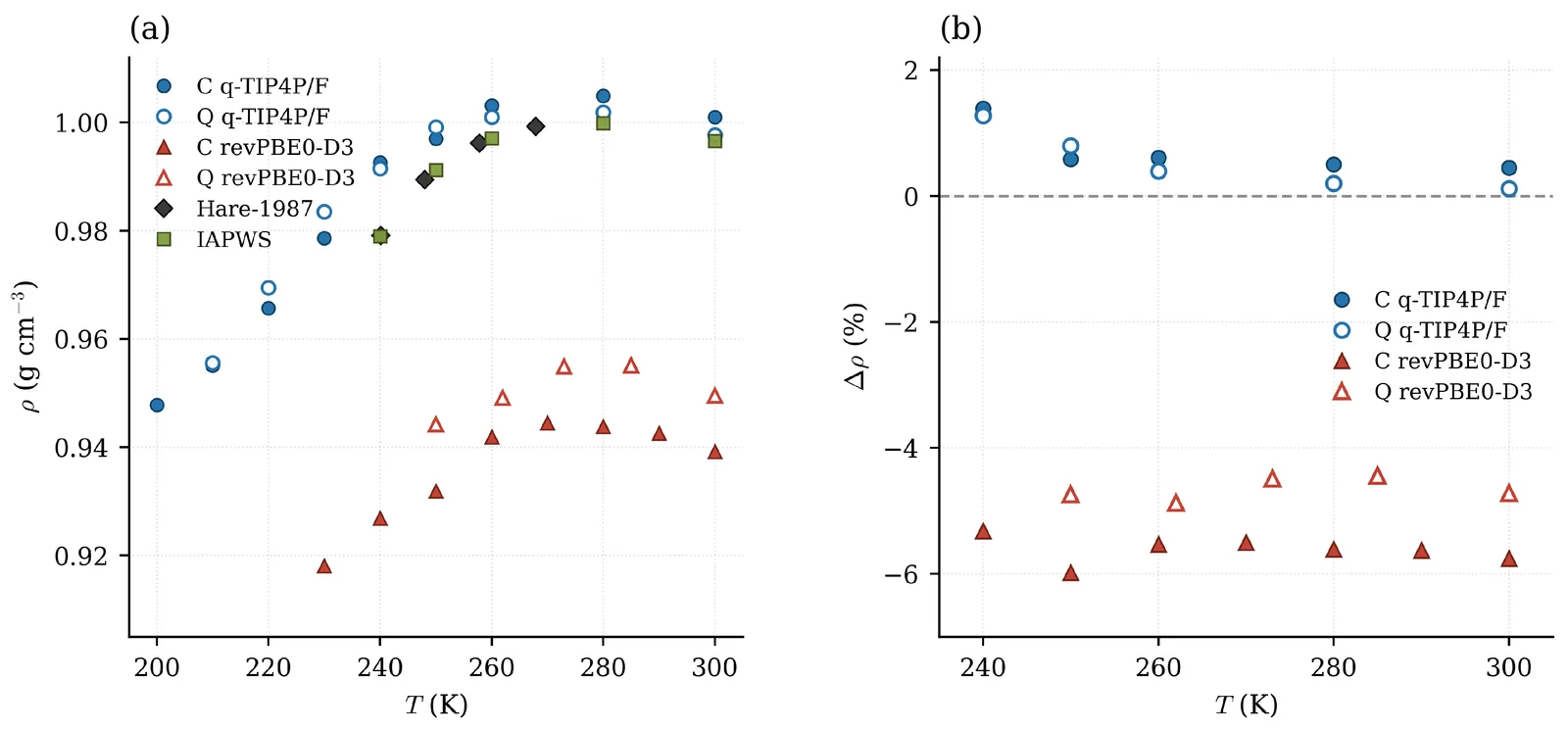
Density of liquid water at 1 bar pressure as a function of temperature. (**a**) Densities from classical- and quantum-nuclei simulations: C q-TIP4P/F denotes classical MD and Q q-TIP4P/F denotes quantum PIMD (P=32) simulations using the q-TIP4P/F water model, both from Ref. [52]; C revPBE0-D3 and Q revPBE0-D3 denote simulations using the DFT revPBE0-D3 functional, sampled through a neural-network surrogate, taken from Ref. [79]; Hare-1987 denotes the experimental density values reported by Ref. [153]; the IAPWS series gives the reference equation-of-state density computed from IAPWS G12-15 [55] at the exact temperatures of the simulations. (**b**) Percentage deviation of the simulated densities in panel (**a**) from the IAPWS G12-15 reference equation of state [55] at the same temperature, computed as $\Delta \rho =100\;\left(\rho_{\mathrm{sim}}-\rho_{\mathrm{IAPWS}}\right)/\rho_{\mathrm{IAPWS}}$, with C and Q denoting the classical and quantum entries, respectively. All data points were digitized from their respective source papers; estimated digitization uncertainty is ±0.0005 g cm^−3^.

Classical-nuclei predictions diverge from experimental observations in several ways at low temperature [22,151]:

**Density Trends:** Within the 200–273 K window at ambient pressure, the nuclear treatment has only a modest effect on the density of liquid water: the intramolecular and intermolecular zero-point contributions act in opposite directions and largely cancel, so classical and path-integral trajectories on the same potential energy surface yield similar densities [52,54]. Figure 4 quantifies this within the window: on the q-TIP4P/F surface, the classical and quantum densities differ by at most 0.005 g cm^−3^, and both lie within 1.4% of the reference equation of state, whereas the revPBE0-D3 surface underestimates the experimental density by 4.4–6.0% under either nuclear treatment. The choice of potential energy surface, and not the nuclear treatment, therefore sets the density error here. Simulations driven by ab initio reference data reach a similar conclusion and locate the temperature of maximum density through the description of dispersion rather than through the nuclear treatment [84]. The near-cancellation is a property of the liquid: for ice I_h_ at 0.1 MPa pressure and on the same q-TIP4P/F surface, classical nuclei overestimate the density by about 4%, while path-integral simulations stay within 0.5% of experiments [151]. The two observations are consistent rather than contradictory: the cancellation that holds in the supercooled liquid is lost once the hydrogen-bond network is frozen, where anisotropic proton delocalization gives less linear hydrogen bonds and lengthens the O⋯O separation of hydrogen-bonded pairs [151]. Both sets of results are model-dependent: they rest on the empirical flexible q-TIP4P/F water model potential rather than on ab initio reference data, and the near-cancellation of NQEs in the liquid is reported as specific to this parametrisation [52].

**Hydrogen Bond Geometry:** Classical first-principles simulations tend to produce overly structured O–H⋯O hydrogen bonds. Path-integral simulations show that NQE-induced proton delocalization broadens the intramolecular O–H peak and lowers the first O–O peak of the radial distribution functions relative to classical nuclei at the same temperature, improving agreement with diffraction data [48]. The added diffuseness of the intramolecular degrees of freedom stretches the covalent O–H bond and raises the root-mean-square molecular dipole moment [48]. This appears in RDF plots as a shift in the intermolecular O–H hydrogen-bond peak to shorter distances, rendering that particular feature more structured even as the first O–O peak is lowered [152].

Experimental evidence for proton delocalization is provided by proton momentum distributions measured by neutron Compton scattering, which path-integral CPMD reproduces in both liquid water and ice [48].

**Radial Distribution Functions:** First-neighbour peaks in $g_{\mathrm{OO}}\left(r\right)$ and $g_{\mathrm{OH}}\left(r\right)$ are sharper in classical simulations than with NQEs, and including quantum effects broadens them toward the experimental values [48]. Early path-integral simulations with empirical potentials already broadened both radial distribution functions—an effect close to that of raising the temperature of a classical simulation—but did not reproduce the shorter momentum tail measured in ice [48]. Path-integral CPMD with a GGA functional lowers the first O–O peak from 3.23 with classical nuclei to 2.84±0.06, closer to the scattering data, and does reproduce the liquid–ice difference in the momentum distribution, although a residual excess of structure remains and is ascribed to the description of exchange, correlation, and dispersion [48]; the apparent accuracy of simple GGA functionals in fact originates from a cancellation of errors due to their incorrect treatment of the anharmonicity of hydrogen bonds [18]. Longer and better-converged ab initio PIMD subsequently showed the net effect on the O–O radial distribution function to be modest, with the first peak lowered but the following minimum deepened and the second peak raised, so that the second coordination shell becomes more structured while the first is mildly de-structured [22].

**Vibrational Spectra:** The O–H stretching frequency is red-shifted in condensed phases due to hydrogen bonding, and at room temperature NQEs themselves produce a red shift of ∼200 cm^−1^ on the O–H stretching peak [18].

### 5.4. Classical Versus Quantum Nuclei: Matched Comparisons with Experiment

The preceding subsections establish that NQEs occur on the energy scale set by $k_{B}T$, and that they influence water structure and hydrogen-bond geometry. It is therefore useful to assess the magnitude of the resulting changes in observables.

Regarding the nuclear isotopic mass, at the ab initio level, Cheng et al. computed the ice I_h_/liquid melting point with classical and with quantum nuclei on the revPBE0-D3 functional, using a neural-network surrogate to make the free-energy integration affordable [79]. Classical equilibrium properties are independent of nuclear mass, so a classical treatment predicts no isotope shift in $T_{m}$ between H_2_O and D_2_O; with quantum nuclei, each isotope’s melting point deviates from its own experimental value by a different amount: 6.2 K for H_2_O against 2.0 K for D_2_O. This results in a predicted isotope shift of 8±2 K against an experimental value of 3.82 K [79].

Eltareb et al. report a comprehensive comparison of classical and quantum simulations against experimental data, for several observables. This study employed the q-TIP4P/F water model, parametrised for path integrals, at P=1 bar and in the temperature window 200≤T<375 K [52]. From it, we can see that PIMD densities agree closely with experiments across both the equilibrium and the supercooled regimes, and the isothermal compressibility exhibits a maximum near 230 K for H_2_O and 235 K for D_2_O, against experimental maxima of 228 K and 233 K, respectively, as reported therein [52]. Diffusion coefficients obtained by RPMD agree well with experiments over the whole range studied [52]. The comparison against classical MD on the same model shows that the isobaric heat capacity, the diffusion coefficient, and the structural quantities change appreciably with the nuclear treatment, whereas the differences are minor for most other properties examined (See Figure 2).

**Figure 5:**
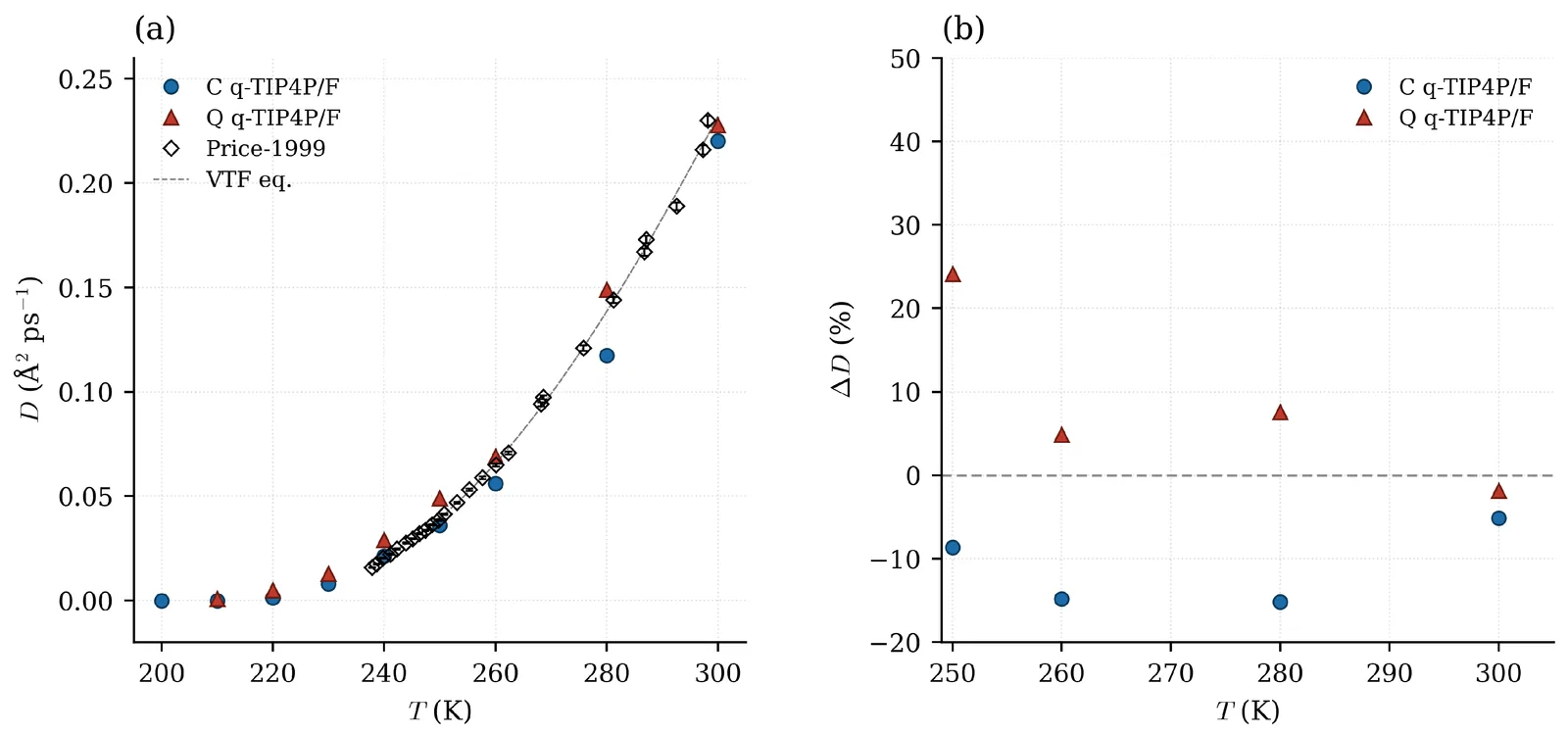
Self-diffusion coefficient of liquid water at 1 bar pressure as a function of temperature. (**a**) C q-TIP4P/F denotes classical MD and Q q-TIP4P/F denotes quantum RPMD (P=32) simulations using the q-TIP4P/F water model, both digitized from Eltareb et al. [52]; Price-1999 denotes the experimental values reported by Ref. [154], with ±1% stated measurement accuracy; VTF eq. is the Vogel–Tammann–Fulcher equation fit of Ref. [154]. (**b**) Relative deviation of the simulated self-diffusion coefficients in panel (**a**) from that same VTF equation, evaluated at the exact simulation temperature and computed as $\Delta D=100\;\left(D_{\mathrm{sim}}-D_{\mathrm{VTF}}\right)/D_{\mathrm{VTF}}$, with C and Q denoting the classical and quantum entries, respectively. All simulated data points were digitized from their respective source papers; estimated digitization uncertainty is $\pm 9\times 10^{-4}$ Å^2^ ps^−1^.

Figure 4 and Figure 5 present comparisons of density and self-diffusion coefficient as a function of temperature. From these, it is clear that the nuclear-quantum correction depends on the observable and the temperature.

**Density:** On the q-TIP4P/F data in Figure 4, the two nuclear treatments differ from each other by at most 0.005 g cm^−3^ over the whole range; the quantum density is the larger of the two at 210–230 K, by up to 0.0049 g cm^−3^ at 230 K, although at 210 K the difference of 0.0005 g cm^−3^ falls within the combined digitization uncertainty of ±0.0007 g cm^−3^ and its sign is therefore not resolved. The sign of the difference is not the same across the 240–250 K interval: the classical point is the larger at 240 K by 0.0011 g cm^−3^, only marginally above the combined digitization uncertainty, and the quantum entry is the larger again at 250 K, by 0.0021 g cm^−3^. At the five temperatures that carry a comparison with the reference equation of state (see Figure 4b), both treatments stay within 1.4% of it [52,55], the largest deviation being +1.39% for classical nuclei at 240 K, and the quantum entry is the closer to the reference at every temperature in the set except 250 K, where the classical treatment is marginally closer (+0.59% against +0.80%). The quantum deviation falls monotonically on heating, from +1.28% at 240 K to +0.12% at 300 K, whereas the classical one does not.

We also include results on the revPBE0-D3 functional [79]. The difference between classical and quantum treatments is about +1 percentage point, slightly larger at 250 K (+1.24) than at 300 K (+1.04), and in both cases this functional underestimates the reference density, by 5.98% and 4.74% at 250 K and by 5.76% and 4.72% at 300 K for classical and quantum nuclei, respectively. Across the whole set, the classical deviation spans −5.32% at 240 K to −5.98% at 250 K, and the quantum one spans −4.44% at 285 K to −4.88% at 262 K. Those two temperatures (250 and 300 K) are the only revPBE0-D3 cases in Figure 4 on which the classical and quantum entries share the same temperature; on the remaining cases, the nearest available quantum point lies 2–10 K away from the classical one, so the classical–quantum difference there is not strictly isothermal, even though each deviation in panel (b) is itself referenced to the equation of state evaluated at the actual temperature of that point.

Panel (b) reports the deviation from the IAPWS G12-15 equation of state for supercooled water [55], computed as $\Delta \rho =100\;\left(\rho_{\mathrm{sim}}-\rho_{\mathrm{IAPWS}}\right)/\rho_{\mathrm{IAPWS}}$ with the formulation evaluated at the exact temperature of each simulated point. This removes the temperature-mismatch error incurred by differencing against the measurements of Ref. [153], which are reported at their own measured temperatures (240.12, 248.03, 257.84, and 267.92 K) and do not coincide with the simulated ones; those measurements are retained in panel (a). No deviation is shown below 240 K, because the simulated points at 230 K and lower fall outside the validity window of the formulation.

**Self-Diffusion:** For the self-diffusion coefficient, Figure 5 reports values computed by Eltareb et al. [52] for the q-TIP4P/F water model with classical and quantum nuclei treatments, compared against reference values from Price et al. [154]. Price et al. fit their results to the Vogel–Tammann–Fulcher equation (Ref. [154]; Equation (4) therein), which formally covers the 242–298 K temperature range, and which is shown in panel (a) of Figure 5 as a dashed grey line. The experimental diffusion coefficients are the 26 individual measurements of Ref. [154] (Table 1), converted from $10^{-9}$ m^2^ s^−1^ to $\;{Å}^{2}$ ps^−1^ and plotted at their own measured temperature, with the stated 1% measurement accuracy shown as error bars. Panel (b) reports the relative deviation from the VTF equation of Ref. [154], $D=D_{0}\exp\left[-B/\left(T-T_{0}\right)\right]$, computed as $\Delta D=100\;\left(D_{\mathrm{sim}}-D_{\mathrm{VTF}}\right)/D_{\mathrm{VTF}}$ with the fit evaluated at the exact simulation temperature.

From Figure 5, we can see that, for the diffusion coefficient, the agreement with the experimental reference varies across the temperature range: quantum nuclei are the closer treatment at 260, 280, and 300 K, whereas at 250 K the classical value is closer; the quantum entries deviate by +24.1% against −8.6% for classical nuclei. At 260 and 280 K, the classical treatment underestimates the reference by about 15%, while the quantum one stays within 8%, and at 300 K both fall within 5% of the reference. Although 300 K lies outside the formal validity range of that reference equation, it is where the closest agreement is found. At the low end, the nearest measured temperature to 230 K lies 7.8 K away—an offset over which *D* itself changes by a factor of two, so the 200–230 K entries appear in panel (a) for completeness rather than as a test of the nuclear treatment.

**Practical Consequences:** In practical terms, the temperature at which a classical treatment stops being a good option depends on which observable is being predicted. A second point follows from comparing the two observables presented here: on q-TIP4P/F, a model parametrised for path integrals, the classical and quantum densities differ by at most 0.005 g cm^−3^—that is, about 0.5% over the range studied [52]—whereas on revPBE0-D3, the same observable shifts by about 1%. The near-cancellation of competing quantum effects on the density is therefore partly a property of the force field and should not be assumed to carry over to another ab initio surface.

These two studies provide an example of where the nuclear treatment is decisive for some observables and marginal for others, within the temperature range of this review. The distinction is not whether a quantity resolves the hydrogen explicitly but whether the competing intramolecular and intermolecular contributions affect that particular observable. The convergence and sampling diagnostics required at these temperatures are presented in Section 3.6 and Table 3.

For the density, the two NQE contributions largely cancel and the nuclear treatment matters comparatively little, consistent with Section 5.1 and Section 5.3. Where they do not cancel, the nuclear treatment is decisive: such is the case of radial distribution functions and proton momentum distributions, where one contribution dominates; isotope effects, which vanish by construction under classical nuclei; activated dynamics such as the self-diffusion coefficient, increasingly so on cooling; and the isobaric heat capacity, into which the intramolecular zero-point energy enters directly. Classical nuclei are therefore a defensible choice in the 200–273 K window when the target is the density and the potential energy surface has itself been validated for that quantity. Classical nuclei are not the optimal choice when the target is a radial distribution function, a proton momentum distribution, an isotope effect, the self-diffusion coefficient, or the isobaric heat capacity. In both studies, the quantum-nuclei result was obtained on an empirical or machine-learned surrogate rather than by direct ab initio path integrals.

## 6. Conclusions and Outlook

Simulating water between 200 and 273 K confronts three coupled challenges: sampling degrades as the dynamics slow; the relative importance of nuclear quantum effects grows on cooling, unevenly across observables; and structural and dynamical observables remain sensitive to the underlying potential energy surface. These cannot be treated in isolation; a reliable low-temperature simulation matches the rigour of the nuclear treatment to the rigour of the electronic structure. In practice, this comes down to two decisions: the propagation scheme, and the electronic-structure method.

*Choosing the Propagation Scheme:* BOMD on a well-converged Born–Oppenheimer surface is the natural baseline whenever NQEs are smaller than the other modelling errors [18,40]. CPMD [45] is competitive in cost per step, but its fictitious-mass dependence and the difficulty of maintaining adiabaticity in the presence of the high O–H stretching frequency make it the weaker choice when low-temperature accuracy is the goal [47]. When the quantum statistics of the nuclei must be captured, imaginary-time path-integral methods (PIMD [49,50], accelerated through PIGLET [53,58] or ring-polymer contraction [57]) are the appropriate tools, with the bead number *P* scaling as $\beta \hbar \omega_{\max}$ and, therefore, rising as temperature falls. Approximate real-time methods (RPMD [70,71]; CMD and its quasi-centroid variant [21,75]) remain the route to vibrational spectra and reaction rates, but each carries characteristic artefacts (spurious resonances in RPMD and curvature-induced frequency shifts in CMD) that are amplified as *T* decreases [70,74].

*Choosing the Functional:* Electronic-structure accuracy cannot be decoupled from the nuclear treatment. Dispersion-corrected GGAs such as revPBE-D3 owe part of their good ambient performance to a cancellation between the functional’s intrinsic error and the neglect of NQEs in classical-nuclei trajectories [121]; this is a fortuitous balance, not evidence of accuracy at 200 K. Meta-GGAs (SCAN, r^2^SCAN) describe intermediate-range dispersion and ice-polymorph energetics well [122,123,124,126] but reproduce ambient liquid structure only when simulated at elevated temperature [127]—a workaround that mimics the missing NQEs rather than supplying them. Hybrid functionals combined with explicit path integrals (e.g., revPBE0-D3 + PIMD) are among the few combinations that quantitatively reproduce both structure and spectroscopy [22,82,136], at a cost that has historically restricted them to short trajectories on small cells.

*Why the Regime is Special:* At ambient conditions, the zero-point energy of the O–H stretch already exceeds $k_{B}T$ by close to an order of magnitude, the bend by a factor of four, and the libration by a factor of under two (Table 5). Cooling from 298 to 263 K lowers $k_{B}T$ by about 12%, so each of these ratios grows: by about 13% for the stretch and the bend, and by 17% for the libration—the mode closest to the classical limit and, therefore, the one whose degree of quantisation changes most on cooling. This matters because the observable that defines the regime is the accelerating growth of tetrahedral ordering on supercooling [26,27], and classical trajectories over-structure liquid water, while including NQEs softens the O–O and O–H radial distribution functions toward experimental values [48,152]. Two methodological consequences follow: Error cancellations calibrated at ambient conditions, such as the compensation between a functional’s intrinsic error and the neglect of NQEs in classical-nuclei trajectories [121], are not guaranteed to hold in the 200–273 K regime, and neither is the practice of raising the simulation temperature to recover ambient liquid structure [127]. At the same time, the cost of treating the nuclei quantum-mechanically rises, since the bead number scales as $\beta \hbar \omega_{\max}$ [18], and the artefacts of approximate real-time methods are amplified as *T* decreases [70,74].

*Where the Field is Heading:* A consequential recent development is the maturation of machine-learning interatomic potentials (MLPs) trained on high-level ab initio reference data [20,77,78,80]. By lowering the per-step cost of a force evaluation by orders of magnitude, MLPs make nanosecond-scale path-integral trajectories feasible, at the accuracy of their underlying ab initio reference, on the system sizes required for nucleation, phase-equilibrium, and amorphous-ice studies [65,79,83,108], and they are now the route by which most quantitative low-temperature results reach the literature. The principal problem is transferability: an MLP trained on ambient data is not guaranteed to hold at 200–273 K, so explicit ab initio validation at the target conditions remains essential. This is complicated by the fact that direct DFT-AIMD benchmarks in the supercooled regime are sparse, so that functional rankings still rest largely on ambient data. We expect MLP-driven, path-integral simulations, trained on hybrid or density-corrected reference data and validated against experimental structural data or higher levels of theory, to become the practical workflow of the coming decade.

*Why It Matters:* Supercooled water is the relevant phase for ice nucleation in mixed-phase clouds and in freezing atmospheric droplets [39,155], for the survival of organs under sub-zero preservation [7,8], for nucleation control in cryopreservation and food processing [10], and for the chemistry that builds complex organic and prebiotic molecules [11,12]. In each setting, prediction demands quantitative agreement with experiments at temperatures where NQEs are non-negligible and direct measurement is fragmented. The convergence outlined above—path-integral sampling driven by machine-learning potentials trained on hybrid-functional reference data—brings these problems within reach of routine simulation at the system sizes and trajectory lengths that the underlying physics demands.

## Figures and Tables

**Table 1:** Crystalline ice phases relevant to the 200–273 K range, including metastable and higher-pressure forms; $r_{OO}$ denotes the position of the first O–O RDF peak (or the nearest-neighbour O–O distance). Superscript letters refer to table notes. Data and notes compiled from neutron and X-ray diffraction studies and related crystallographic or equation-of-state sources.

Phase	Crystal System	ρ (g cm^−3^)	*T* (K)	*P* (atm)	$r_{\mathrm{OO}}$ (Å)	H-Order	Refs.
Ice I_h_	Hexagonal	0.92	273	1	2.71 ^a^	Disordered	[30,31,32]
Ice I_c_	Cubic	0.93	130–250 ^b^	1	2.76	Disordered	[33,34,35]
Ice I_sd_	Trigonal	—	<200	1	—	Disordered	[34,36]
Ice II	Rhombohedral	1.19–1.20	180–240	1974–4935 ^c^	2.80	Ordered	[37,38]
Ice III	Tetragonal	1.16–1.18	240–251	2063–3454 ^c^	—	Disordered	[38]

^a^ Bergmann et al. report the distribution of nearest-neighbour O–O distances for polycrystalline ice I_h_ at −16.8 °C; the peak position is 2.71 Å (mean distance 2.76 Å). ^b^ Stability/observation range for ice I_c_ from [34]; crystal system, density, and pressure from [33]. ^c^ Stability-field pressures converted from MPa data in the source; 1 atm $\approx 1.013\times 10^{-1}$ MPa.

**Table 2:** Comparison of AIMD methods for water simulations and an important parameter to be converged for each. Other parameters may require convergence depending on the method. Cost entries are formal order-of-magnitude scalings relative to BOMD with a GGA functional, not benchmarked wall-clock comparisons.

Method	Nuclei	Cost	Main Limitation	Recommended Use	What to Converge or Check
BOMD	Classical	1× ^a^	Neglects NQE	Baseline when NQEs are not critical; forces on the converged Born–Oppenheimer surface [18,40]	SCF tolerance and time step; drift of the conserved quantity [40]
CPMD	Classical	<1×	Fictitious-mass choice may introduce systematic force biases	Fast per step, avoiding explicit SCF minimisation; only when classical nuclei are acceptable [40,47]	Fictitious electronic mass; validate against a BOMD reference [47]
PIMD	Quantum	≥32×	Expensive; *P* must increase as *T* decreases	Rigorous NQE; benchmark and validation when highest equilibrium accuracy is required [18]	*P* converged at the target temperature, not carried over from 300 K [49,81]
PIGLET	Quantum	≥6×	Requires a tailored GLE thermostat on ring-polymer modes	Faster NQE convergence; production NQE simulations at reduced cost [53,58]	GLE fitted to the target *T* and frequency range; estimators converge at different *P* [53,58]
RPC	Quantum	<P×	Changes cost, not configurational sampling	Reduces the replicas carrying the expensive potential component [57]	Contracted forces must reproduce the uncontracted result for the target observable [57,82]
RPMD	Quantum	∼P×	Approximate dynamics; spurious peaks in vibrational spectra	Kubo-transformed real-time correlation functions; diffusion coefficients and reaction rates [70,71]	Interpretation of real-time correlation functions [70,71]
CMD	Quantum	∼P×	Curvature problem at low *T*: artificial broadening and red shift of the O–H stretch	Vibrational spectra of liquid water at moderate/high *T*; avoid low-*T* liquid and ice without QCMD-type corrections [21,74,75]	Suitability at the target temperature [74,75]
MLP	Host method	≪1× per force	Transferability outside the training distribution not guaranteed	Replaces the PES; makes nanosecond path-integral trajectories affordable [77,78,79]	Coverage of the training set in *T* and density; validate against ab initio data at the target conditions [83]

^a^ Reference cost; all other entries are formal scalings only.

**Table 3:** General convergence and sampling criteria for molecular dynamics of water below 273 K, applying to every method.

Parameter	What to Check	Criterion/Reported Evidence	Ref.
Equilibration	Treat the target observable as a time series; discard the initial transient	Production averages must be insensitive to the choice of discard point; short equilibration times risk mistaking a metastable state for true equilibrium at low temperature	[91,92,93]
Relaxation/ autocorrelation time	Autocorrelation function of the observable; statistical inefficiency	The α-relaxation time from the self-intermediate scattering function is the reference timescale (see Section 3.6)	[87,88,92]
Trajectory length	Production time relative to the slowest relevant correlation time	Must be long enough for the block-averaged error to plateau; near 200 K, several nanoseconds may be insufficient	[94]
Independent replicas	Repeat from independently prepared starting configurations	Replica-to-replica scatter should not exceed the within-replica uncertainty; disagreement indicates trapping in a single basin	[92]
Statistical error	Block averaging or an equivalent treatment of correlated data	The naive standard error underestimates uncertainty when samples are correlated	[89,92]
Finite size	Cell size against the range of the correlations of interest; size correction to transport coefficients	Diffusion coefficients and viscosities from periodic cells are system-size-dependent; studies of this regime with ab initio-quality potentials have been confined to roughly 250–500 molecules	[93,94,95]
Incipient crystallisation	Per-frame classification with local bond-orientational order parameters or CHILL+	Distinguishes a supercooled liquid from a partially crystallised sample; a usable window exists (see text)	[90,96,97,98]
Cooling-rate hysteresis	Report the cooling rate; check for hysteresis on reheating	Properties of glassy states obtained by cooling depend on the rate applied	[99]

**Table 4:** Structural and thermodynamic validation targets for molecular dynamics of water in the 200–273 K range.

Observable	What to Compare	Reference Point	Ref.
Structure factor S(Q)	Compare in reciprocal space before Fourier transforming; position and height of the first diffraction peak and its shift on cooling	X-ray and neutron measurements of water around the compressibility minimum; OO, OH, and HH partial structure factors reported from 220 K upwards	[26,100]
Partial RDFs	Observe $g_{\mathrm{OO}}$, $g_{\mathrm{OH}}$, and $g_{\mathrm{HH}}$; positions and heights of the first intramolecular and intermolecular peaks	Site–site functions from empirical potential structure refinement of neutron data over 220–673 K; quantum nuclei broaden the first peaks of $g_{\mathrm{OH}}$ and $g_{\mathrm{HH}}$ substantially while only mildly de-structuring $g_{\mathrm{OO}}$	[79,100,104]
O–O coordination number	Value at the first minimum of $g_{\mathrm{OO}}\left(r\right)$, and the position of the isosbestic point	Temperature-independent isosbestic point near 3.3 Å in X-ray diffraction on supercooled water	[26]
Tetrahedral order parameter *q*	Mean value and the full distribution; *q* growth on cooling	*q* defined over the four nearest neighbours; the accelerating tetrahedral ordering on supercooling is the observable that defines this regime	[26,27,105,106]
Intermediate-range order	Height and position of the 4th and 5th peaks of $g_{\mathrm{OO}}\left(r\right)$ near 9 and 11 Å as a function of temperature	These correlations continue to strengthen on supercooling down to 234.8 K	[27]
Ring statistics	Distribution of hydrogen-bond ring sizes in the water structure; the counting scheme must be stated alongside the result	Different counting schemes yield different topologies for the same configurations and are complementary rather than equivalent	[102]
Isobaric heat capacity $C_{P}$	Magnitude and temperature of the $C_{P}$ maximum on cooling at 1 bar	Maximum of about 218 J mol^−1^ K^−1^ at 229 K from ultrafast calorimetry on evaporatively cooled microdroplets. In simulation, $C_{P}$ is one of the few quantities for which classical and path-integral trajectories on the same model differ appreciably	[52,103]

**Table 5:** Vibrational frequencies of liquid water and the corresponding H-atom zero-point energies expressed relative to the thermal energy $k_{B}T$. Abbreviations: *str* = intramolecular O–H stretching mode ($\nu_{S}$); *bend* = intramolecular H–O–H bending mode ($\nu_{2}$); *libr* = intermolecular librational (hindered-rotation) mode ($\nu_{L}$). The 263–298 K rows are for liquid water measured by FTIR with a laboratory micro-spectrometer over 263–363 K [147]. No row below 263 K is available because that source series does not extend lower, so the trend is shown over the range for which liquid-phase measurements of all three bands exist. ${\tilde{\nu }}_{\mathrm{str}}$ is the intermediate-coordination (IW) Gaussian sub-band peak position, and ${\tilde{\nu }}_{\mathrm{libr}}$ is the main librational frequency obtained in that source from an empirical ν–*T* relationship rather than by direct measurement. ZPE is computed as $\frac{1}{2}hc\tilde{\nu }$ per mode from the frequency in the same row; $\Lambda_{H}$ is the free-particle thermal de Broglie wavelength of hydrogen, $\Lambda_{H}={\left(2\pi \hbar^{2}/m_{H}k_{B}T\right)}^{1/2}$ [146].

	$k_{B}T$	${\tilde{\nu }}_{\mathrm{str}}$	$\frac{\mathrm{ZPE}(\mathrm{str})}{k_{B}T}$	${\tilde{\nu }}_{\mathrm{bend}}$	$\frac{\mathrm{ZPE}(\mathrm{Bend})}{k_{B}T}$	${\tilde{\nu }}_{\mathrm{libr}}$	$\frac{\mathrm{ZPE}(\mathrm{libr})}{k_{B}T}$	$\Lambda_{H}$
*T* (K)	(kcal mol^−1^)	(cm^−1^)		(cm^−1^)		(cm^−1^)		(Å)
298	0.59	3462.0	8.36	1652.7	3.99	717.6	1.73	1.01
273	0.54	3454.0	9.10	1648.8	4.34	732.7	1.93	1.05
263	0.52	3464.1	9.48	1648.8	4.51	738.7	2.02	1.07

## Data Availability

No new data were created or analyzed in this study. Data sharing is not applicable to this article.

## Abbreviations

The following abbreviations are used in this manuscript:

AIMD	Ab Initio Molecular Dynamics
BOMD	Born–Oppenheimer Molecular Dynamics
CMD	Centroid Molecular Dynamics
COM	Complex Organic Molecule
CPMD	Car–Parrinello Molecular Dynamics
DFT	Density Functional Theory
FTIR	Fourier-Transform Infrared Spectroscopy
GGA	Generalized Gradient Approximation
GLE	Generalized Langevin Equation
GPW	Gaussian and Augmented Plane Waves
HDL	High-Density Liquid
IAPWS	International Association for the Properties of Water and Steam
LDL	Low-Density Liquid
MD	Molecular Dynamics
MLP	Machine-Learning Interatomic Potential
NQE	Nuclear Quantum Effects
PES	Potential Energy Surface
PIGLET	Path-Integral Generalized Langevin Equation Thermostat
PIMD	Path-Integral Molecular Dynamics
QCMD	Quasi-Centroid Molecular Dynamics
RDF	Radial Distribution Function
RPC	Ring-Polymer Contraction
RPMD	Ring-Polymer Molecular Dynamics
SCF	Self-Consistent Field
TMD	Temperature of Maximum Density
TRPMD	Thermostatted Ring-Polymer Molecular Dynamics
ZPE	Zero-Point Energy
